# Ecological Segregation in Space, Time and Trophic Niche of Sympatric Planktivorous Petrels

**DOI:** 10.1371/journal.pone.0062897

**Published:** 2013-04-30

**Authors:** Joan Navarro, Stephen C. Votier, Jacopo Aguzzi, Juan J. Chiesa, Manuela G. Forero, Richard A. Phillips

**Affiliations:** 1 Institut de Ciències del Mar (ICM-CSIC), P. Marítim de la Barceloneta, Barcelona, Spain; 2 Marine Biology & Ecology Research Centre, Plymouth University, Plymouth, United Kingdom; 3 Laboratorio de Cronobiología, Departamento de Ciencia y Tecnología, Universidad Nacional de Quilmes - CONICET, Buenos Aires, Argentina; 4 Departamento de Biología de la Conservación, Estación Biológica de Doñana (EBD-CSIC), Sevilla, Spain; 5 British Antarctic Survey, Natural Environment Research Council, High Cross, Cambridge, United Kingdom; Liverpool John Moores University, United Kingdom

## Abstract

The principle of competitive exclusion postulates that ecologically-similar species are expected to partition their use of resources, leading to niche divergence. The most likely mechanisms allowing such coexistence are considered to be segregation in a horizontal, vertical or temporal dimension, or, where these overlap, a difference in trophic niche. Here, by combining information obtained from tracking devices (geolocator-immersion and time depth recorders), stable isotope analyses of blood, and conventional morphometry, we provide a detailed investigation of the ecological mechanisms that explain the coexistence of four species of abundant, zooplanktivorous seabirds in Southern Ocean ecosystems (blue petrel *Halobaena caerulea*, Antarctic prion *Pachyptila desolata*, common diving petrel *Pelecanoides urinatrix* and South Georgian diving petrel *P. georgicus*). The results revealed a combination of horizontal, vertical and temporal foraging segregation during the breeding season. The stable isotope and morphological analyses reinforced this conclusion, indicating that each species occupied a distinct trophic space, and that this appears to reflect adaptations in terms of flight performance. In conclusion, the present study indicated that although there was a degree of overlap in some measures of foraging behaviour, overall the four taxa operated in very different ecological space despite breeding in close proximity. We therefore provide important insight into the mechanisms allowing these very large populations of ecologically-similar predators to coexist.

## Introduction

Analyses of ecological segregation seek to explain how species or populations differ in their use of limited resources [1]. According to the principle of competitive exclusion, ecologically-similar species are expected to partition their use of resources, leading to niche divergence [1], [2]. Hence, character displacement may allow closely-related species to coexist in sympatry [3]. The most likely underlying ecological mechanisms are considered to be inter-specific segregation in one or more horizontal, vertical or temporal dimensions, or a difference in trophic niche [4], [5]. Identifying the most important factors underlying segregation in different environments is therefore a key goal of ecological studies.

An enduring problem for many air-breathing marine vertebrates, such as seabirds, which breed on land in large colonies, is how to locate sufficient marine resources for maintenance and reproduction. All seabirds are central-place foragers during the breeding season, with foraging ranges constrained by the distribution of prey in three-dimensional space (i.e., vertical, horizontal and temporal) [6]–[11]. Given their patchy geographic distribution, islands that are free from terrestrial predators often hold mixed-species breeding colonies composed of thousands to millions of individuals [12], [13]. Under these conditions, competition for resources is likely to be particularly intense, and colonial seabirds provide clear examples of ecological segregation by a variety of mechanisms that are presumed to reduce inter-specific competition for food [14]–[16]. Nevertheless, in some communities, a lack of resource partitioning appears to arise because of a superabundance of prey [17]. Therefore, much remains to be learnt about the role of niche partitioning among colonial seabirds.

The burgeoning use of bio-logging technology has greatly improved our knowledge of the foraging behaviour of seabirds and consequently how they avoid competition for food [18], [19]. Until recently, tracking devices were large relative to animal body-size, and consequently most studies were conducted on medium to large species (>500 g). However, in terms of the number of individuals, many seabird communities are dominated by small species. For example, in the sub-Antarctic, small petrels consume ∼1 million tonnes of crustaceans per year, mainly Antarctic krill *Euphausia superba* [13], [20]. Their dependence on similar prey has led to speculation that inter-specific competition could be a fundamental mechanism structuring small petrel communities [21]–[23]. Conversely, because of the huge biomass of krill in the Southern Ocean and the removal by hunting of large baleen whales (species of the suborder Mysticeti) that were formerly major consumers, the possibility exists that krill availability is not limiting and that other mechanisms explain ecological isolation. According to niche theory, these species should show divergent foraging strategies to avoid competition for the same resources [6], [24].

In the present study, we investigated the ecological mechanisms that may explain the coexistence of four small (120–200 g), very abundant, zooplanktivorous seabirds - blue petrel (*Halobaena caerulea*), Antarctic prion (*Pachyptila desolata*), common diving petrel (*Pelecanoides urinatrix*) and South Georgian diving petrel (*P. georgicus*) - which breed in sympatry on islands in the Southern Ocean. Previous analyses of stomach contents indicate that although the diets of these four species are composed primarily of crustaceans, Antarctic prions and blue petrels tend to eat more Antarctic krill, other euphausiids and fish, whereas common and South Georgian diving petrels consume more copepods [25]–[29]. Differences in stable isotope ratios were attributed to potential segregation in foraging grounds [22], . In addition, deployment of capillary tube depth gauges suggested that there were substantial differences in dive depth among these four species [27], [28], [30]–[33]. However, there are no accurate data published on distribution, dive characteristics or behaviour of individuals tracked at sea. We analysed spatial movements, diving strategies and at-sea diel activity patterns of each species by using miniaturized geolocator-immersion loggers (also termed Global Location Sensor or GLS-immersion loggers) and time depth recorders (TDRs) to test for segregation in horizontal, vertical and temporal axes. We also investigated segregation among species in terms of stable isotope ratios, as a measure of trophic divergence. In addition, we measured wing morphology to test for potential functional relationships between flying and diving capacities, as well as foraging strategies.

## Materials and Methods

### Ethics Statement

Device deployment took <3 minutes and on no occasion did it interfere with reproduction or have an apparent deleterious effect on the study animals [34]. All work was approved by the Ethics Committee of the British Antarctic Survey and carried out under permit from the Government of South Georgia and the South Sandwich Islands.

### Study Area and Species

**Figure 1:**
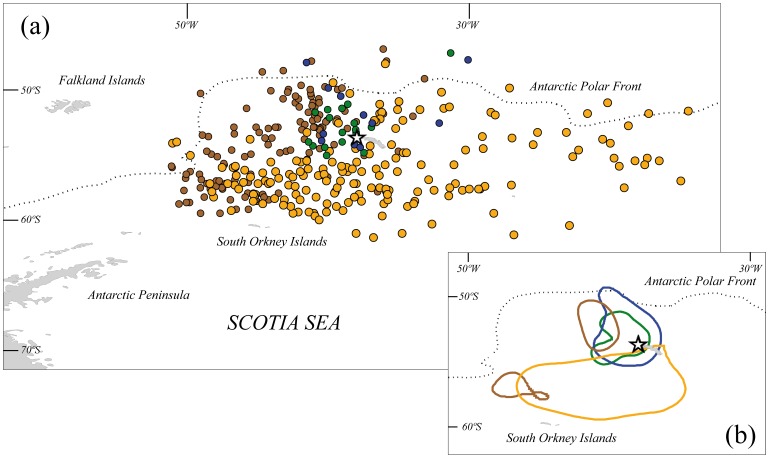
Spatial distribution of foraging areas. (a) Locations and (b) core areas (50% kernel contours) of 10 Antarctic prions (brown symbols & contours), 12 blue petrels (orange symbols & contours), 9 common diving petrels (blue symbols & contours) and 8 South Georgian diving petrels (green symbols & contours) tracked using geolocators during incubation at Bird Island, South Georgia in summer 2010/11. The white star indicates the position of the colony.

**Figure 2:**
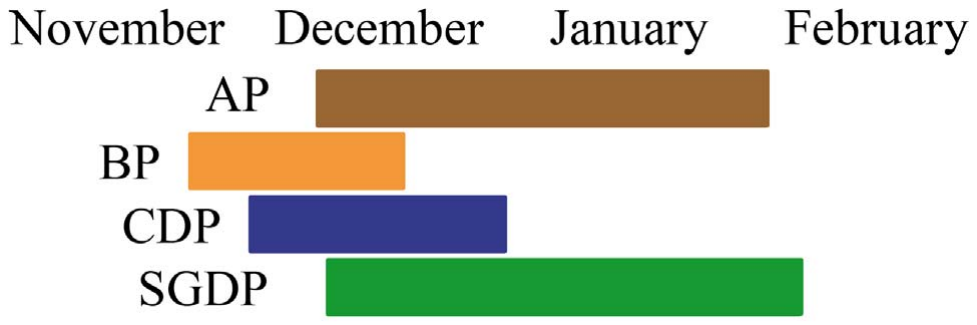
Incubation period of the four small petrels in Bird Island during austral summer 2010/11. ****Antarctic prion (AP), blue petrel (BP), common diving petrel (CDP) and South Georgian diving petrel (SGDP).

Fieldwork was carried out at Bird Island, South Georgia (54°00’S, 38°03’W; Fig. 1) during the austral summer (November 2010 to February 2011), when the incubation period overlaps between the four study species (Figure 2). Burrows of South Georgian diving petrels are found mainly in fine scree slopes and moss areas, whereas those of the other three species are in slopes covered by tussock grass (*Poa flabellata*) [35]. These four species show the typical Procellariiform pattern of a single-egg clutch and slow chick development, with both parents sharing incubation and chick-rearing duties [13]. These species are considered to be sexually monomorphic, and males and females show similar feeding behaviour [13], [23]; therefore, sex was not included in our analysis.

### Instrument Deployments and Analysis

In order to study at-sea behaviour, we attached leg-mounted GLS-immersion loggers (MK18 model, 1.5 g, developed by British Antarctic Survey, Cambridge, UK) to 10 Antarctic prions, 12 blue petrels, 9 common diving petrels and 8 South Georgian diving petrels. Approximate locations were derived from light data using established geolocation methods [36]. In addition, the loggers measure saltwater immersion every 3 sec. and record the number of positive tests at 10 min. intervals (providing a value between 0 and 200 reflecting the proportion of time spent on the water, rather than in flight or on land). Diving behaviour was investigated by equipping 19 common diving petrels, 9 South Georgian diving petrels, 3 Antarctic prions and 5 blue petrels with miniaturized TDRs (Cefas G5, 2.5 g, less than 1 g in water; Cefas Technology Ltd, Lowestoft, UK). TDRs were programmed to record pressure (depth) every 1 sec and temperature every 1 h. Depth and temperature were recorded with a resolution of 0.2 m and 0.1°C, respectively. The TDR was attached to the central tail feathers using waterproof tape to preserve the integrity of the plumage. We used a burrow-scope (with an infra-red light source to avoid disturbance) on daily visits to determine the exact day when equipped birds started and ended the foraging trip (to determine the duration of each foraging trip). After a single foraging trip, birds were recaptured in their burrow and the logger removed. Handling times during device deployment and retrieval were <5 min., and birds were always returned to their burrows.

### Stable Isotope Analysis

Trophic overlap/divergence was assessed by comparing δ^15^N and δ^13^C stable isotope values in red blood-cells taken from 15 adults of each species at the end of incubation. δ^15^N reflects trophic status, whereas δ^13^C indicates carbon source or foraging distribution [37]. We took 0.2 ml of blood from the brachial vein, and centrifuged samples within 2 hours to separate red cells and serum. Red cells were stored frozen (−20°C) until stable isotope analyses at the Laboratory of Stable Isotopes at the Estación Biológica de Doñana (www.ebd.csic.es/lie/index.html). All samples (about 0.9–1 mg) were combusted at 1020°C using a continuous flow isotope-ratio mass spectrometry (Europe Scientific, UK) system by means of a Carlo Erba 1500 N C elemental analyser interfaced with Delta Plus CL mass spectrometer. All isotope abundances are expressed in δ-notation as parts per thousand (‰) deviation from the IAEA standard AIR (δ^15^N) and VPDB (δ^13^C). Based on lab standards, the measurement error was ±0.2 and ±0.1 for δ^15^N and δ^13^C, respectively. For small birds, the isotope ratios in red blood cells integrate the diet of the previous 3–5 weeks [38]; thus, stable isotope ratios in the sampled petrels represented the incubation period.

### Wing Morphology

We calculated wing loading and aspect ratio of the four Procellariiformes to provide a measure of flight performance [39]. Fifteen breeding adults of each species (different to those used for tracking or blood sampling) were captured during incubation, weighed, wingspan (distance between wing tips, with wings at full stretch) was measured, and a digital picture taken of the right wing (with a reference scale). Wing area (area of both wings including the part of the body between the wings) was estimated from the digital picture using Image J software (version 1.30, http://rsb.info.nih.gov/ij). Wing loading (N·m^−2^) was calculated as body weight [i.e. body mass (kg) · (9.8 m·s^−2^)] divided by wing area (m^2^). Aspect ratio (dimensionless) was calculated as wingspan (m) squared divided by wing area (m^2^) [39].

### GLS Data Analysis

Geolocation can provide two positions per day, with a mean error of approximately 186±114 km [36]. Light data were analyzed using the BASTrak software suite; dawn and dusk transition times were determined from light curves, and latitude and longitude estimated from day length and the time of local mid-day relative to Greenwich Mean Time, respectively. We assumed a sun elevation angle of −3.5°, based on known positions obtained during calibration periods before and after each deployment. Unrealistic positions (those resulting from interference to light curves at dawn or dusk) were removed from further analyses. Deployments took place outside equinox periods and hence avoided associated problems with latitude estimation [36].

### Diel at-sea Activity Patterns

Time series periodicity was screened by Fourier analysis, considering the potential occurrence of uni- or bimodal activity profiles [40]. A modelled cosenoidal function with a period of 24 h (i.e., the so called “fundamental harmonic”), and its first entire submultiple of this fundamental (12h) were square fitted onto the time series to obtain a Power Content value (PC24 h or PC12 h, respectively) as a measure of the goodness of fit (i.e. statistical significance for p<0.05). The Power Content values represent the percentage of variance explained by each modelled function (PC24 or PC12) with respect to the total variance of the time series. The PC12 h/PC24 h ratio was used to assess the relevance of the 12 h *versus* 24 h harmonic as a proxy of differences in the relative importance of diurnal or crepuscular behaviour in each species. Only time series with significant periodicity (in previous Fourier screening) were included in the next waveform analysis, seeking to examine niche differences related to variation in timing of maximum activity.

A 24 h waveform analysis was carried out in order to depict the timing of maximum at-sea-activity (immersion). Individual waveforms were constructed by subdividing the data sets into subsets of 24 h that were averaged (± standard error) at corresponding temporal bins. The temporal activity peak limits (i.e. the onset and offset) were identified by the Midline Estimated Statistic of a Rhythm (MESOR); this parameter was calculated by averaging all waveform values, and is represented as a horizontal threshold line on all the waveform plots. Waveform values above the MESOR indicated the significant increment in immersion activity. In cases of multiple peaks, appearance in waveforms of the timing of the first and the second highest bouts were reported. A global and distinctive diel activity pattern for each species was obtained by averaging the waveforms of different individuals.

The percentage of activity (i.e., mean values above the MESOR) was calculated as a proxy of rhythm strength. In addition, the diurnal and nocturnal distribution of at-sea-activity of each animal was calculated by considering the following ratio: activity during daylight in relation to the total activity during the 24 h. The clustering of activity phases obtained from waveforms was compared using Rayleigh z-tests [41]. This test gives the significance in the clustering of individual phases distributed in circular coordinates (e.g., 24 h). Also, the mean phase of each activity bout can be obtained by calculating the vectorial mean of the individuals (i.e., the *r* vector), together with confidence limits. Activity data were analysed using the statistical tools included in El Temps software (A. Díez-Noguera, University of Barcelona, Spain).

### Dive Analysis

Downloaded TDR data were processed using DiveMove 1.2.6 software [42], available through GNU R (R Development Core Team 2007). Pressure data were corrected for surface drift (zero offset correction; [43]) and a number of parameters were automatically extracted for each dive (see Table 1). Specifically, we calculated the dive rate (number of dives per hour), dive depth (average of the mean dive depth of each individual), maximum dive duration (average of the maximum dive depth reach for each individual), dive duration (average of the mean dive duration of each individual) and maximum dive duration (average of the maximum dive duration reach for each individual). Dive threshold was set at 1 m depth to exclude the effect of wave action when birds were on the water surface.

**Table 1:** Trip duration and diving characteristics (mean and standard deviation) of common diving petrels (CPD), South Georgian diving petrels (SGDP), Antarctic prions (AP) and blue petrels (BP) tracked with TDRs and GLS-immersion loggers during incubation at Bird Island, South Georgia, in summer 2010/11.

	CDP		SGDP		AP		BP		ANOVA results		
	mean	SD	mean	SD	mean	SD	mean	SD	*F*	*Dfd*	*P*
Trip duration-GLS (days)	1.2_A_	0.4	1.7_B_	0.5	6.7_C_	1.1	7.1_C_	0.9	136.71	39	<0.0001
Trip duration-TDR (days)	1.1_A_	0.4	2.1_B_	0.1	5.9_C_	1.2	5.3_C_	1.5	72.61	30	<0.0001
Dive rate (dives·h^−1^)	36.14_A_	13.77	9.02_B_	1.76	0.26_ C_	0.16	0.09_C_	0.07	38.13	30	<0.0001
Dive depth (m)	1.84_A_	0.81	3.69_B_	0.96	1.67_C_	0.09	1.85_C_	0.29	9.93	30	<0.0001
Maximum dive depth (m)	10.69_A_	4.57	18.08_B_	3.65	3.48_C_	1.03	3.69_C_	0.75	11.01	30	<0.0001
Dive duration (s)	10.91_A_	4.85	14.98_A_	3.13	2.19_B_	0.33	3.45_B_	1.04	11.32	30	<0.0001
Maximum dive duration (s)	37.32_A_	8.57	44.17_A_	5.91	6.67_B_	2.08	8.41_B_	2.07	38.86	30	<0.0001

Denominator degrees of freedom (*dfd*) are provided. Numerator degree of freedom = 3 for all parameters. The results of post-hoc Tukey HSD test are shown by the subscripts: for each variable – the means of species with the same letter were not significantly different.

### Statistical Analyses

We compared the foraging trip duration of birds with GLS loggers or TDRs, dive characteristics (dive rate, dive depth, maximum dive depth, dive duration and maximum dive duration), stable isotope ratios (δ^15^N and δ^13^C), body mass, and flight performance metrics (wingspan, wing loading and aspect ratio) between the four small petrel species (AP, BP, CDP and SGDP) by using ANOVA and Tukey post-hoc tests (the independent variable was species, and dependent variables were the trip, dive or flight characteristics, or isotope ratios). Any dependent variable that was not normally distributed (trip duration, dive duration and aspect ratio) was log_10_ transformed prior to statistical analysis. All statistical analyses were conducted in PASW 18.0 software (SPSS, Inc., Chicago, Illinois). The significance level was set at P = 0.05.

## Results

### Foraging Trip Duration and Spatial Distribution

All GLS and TDR loggers were recovered and downloaded successfully. Foraging trip duration during incubation for birds tracked using GLS-immersion loggers or TDRs differed significantly among species (Table 1). In particular, pairwise Tukey post-hoc test indicated that the mean foraging trip duration was highest (p<0.05) in blue petrel, followed by Antarctic prion, South Georgian diving petrel and common diving petrel (both diving petrels showed similar foraging trip duration; Tukey post-hoc tests, p>0.05, Table 1). All species foraged predominantly to the south of the Antarctic Polar Front in Antarctic waters, and there was evidence of spatial segregation between some species (Fig. 1). Antarctic prions foraged to the west of the South Georgia archipelago, with one core area (50% kernel) close to the colony, and another at higher latitude to the northwest of the South Orkney Islands. Although there was some overlap (3%) in the latter area, the distribution of blue petrels was more extensive, concentrated to the south and southwest of South Georgia, although also extending (at lower densities) to the east. Common and South Georgian diving petrels foraged close to and predominantly to the west of, the breeding colony, and overlapped almost entirely with one another. Both species of diving-petrel were largely segregated at sea from both Antarctic prions and blue petrels.

### Diving Behavior

**Figure 3:**
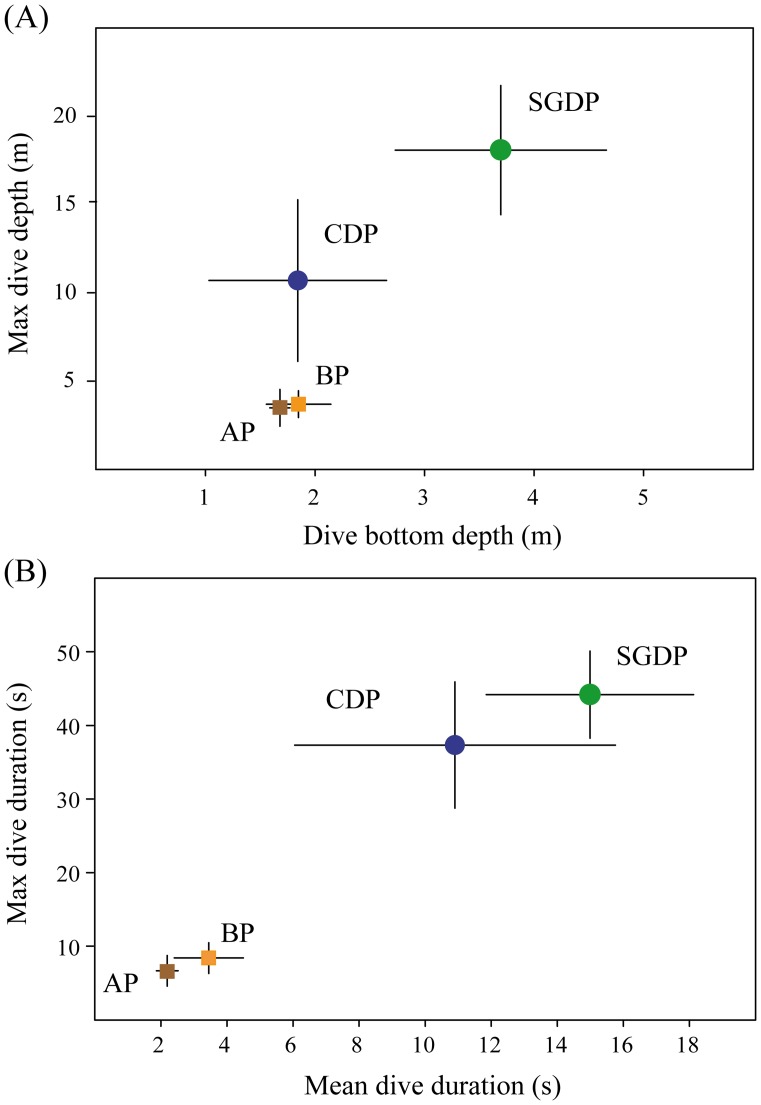
Diving capability descriptors. Mean and standard deviation of (a) maximum dive depth and bottom dive depth, and (b) maximum dive duration and mean dive duration of 19 common diving petrels (CDP), 6 South Georgian diving petrels (SGDP), 3 Antarctic prions (AP) and 5 blue petrels (BP) fitted with TDRs during incubation at Bird Island, South Georgia in summer 2010/11.

**Figure 4:**
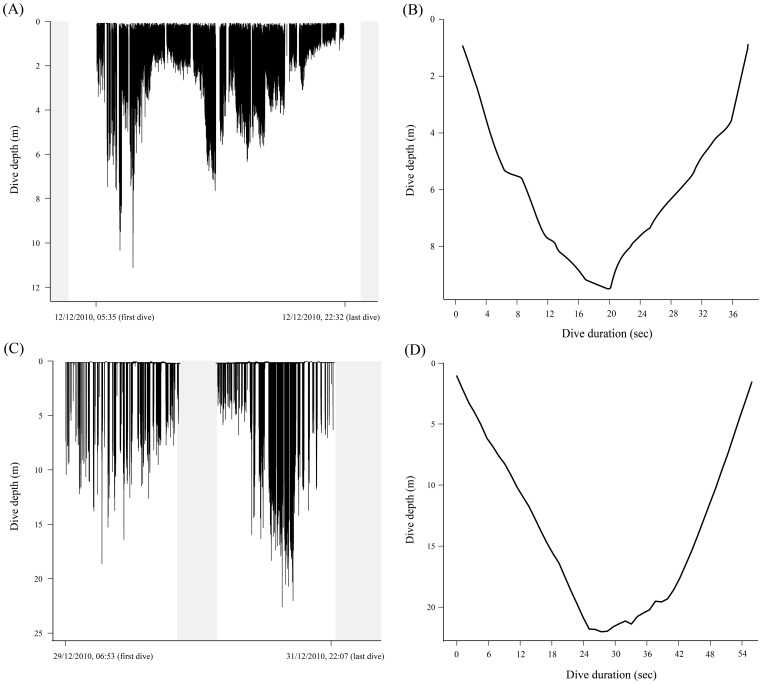
Example of the diving activity and a dive event of a common and a South Georgian diving petrel. Diving activity during the entire foraging trip and one dive of common diving petrel [(a) and (b), respectively] and South Georgian diving petrel [(c) and (d), respectively] measured with TDRs during incubation at Bird Island, South Georgia in summer 2010/11. Shaded areas indicate darkness.

**Figure 5:**
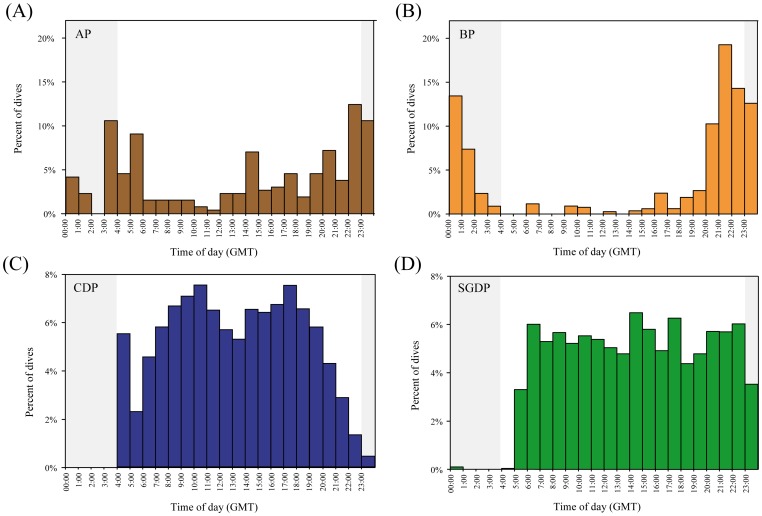
Temporal diving activity patterns. Diel patterns in dive frequency of birds fitted with TDRs loggers during incubation at Bird Island, South Georgia in summer 2010/11. Shaded areas indicate darkness. Species abbreviations: AP (Antarctic prion), BP (blue petrel), CDP (common diving petrel) and SGDP (South Georgian diving petrel).

The rate of dives per hour (dive rate) differed between species, and was four times greater for common diving petrels than for South Georgian diving petrels (Tukey post-hoc tests; p<0.0001), and in both diving petrel species was far higher (p<0.0001) than for Antarctic prions and blue petrels which did not differ in the dive rate (p>0.05; Table 1). Mean bottom depth differed between species (Table 1), and was greatest in South Georgian diving petrel (Tukey post-hoc tests; p<0.0001), and similar in the other three species (p>0.05); however, most dives conducted by Antarctic prion and blue petrels were shallow. In contrast, mean maximum dive depth, and mean and maximum dive durations were greatest for South Georgian than common diving petrels (Tukey post-hoc tests; p<0.0001), followed by Antarctic prions and blue petrels which dived for much less time and reached much shallower depths (Table 1, Fig. 3 and 4). The frequency of dives of Antarctic prions and blue petrels were highest during darkness, whereas nearly all dives of common and South Georgian diving petrels occurred during daylight (Fig. 5).

### Diel at-sea Activity Patterns

**Figure 6:**
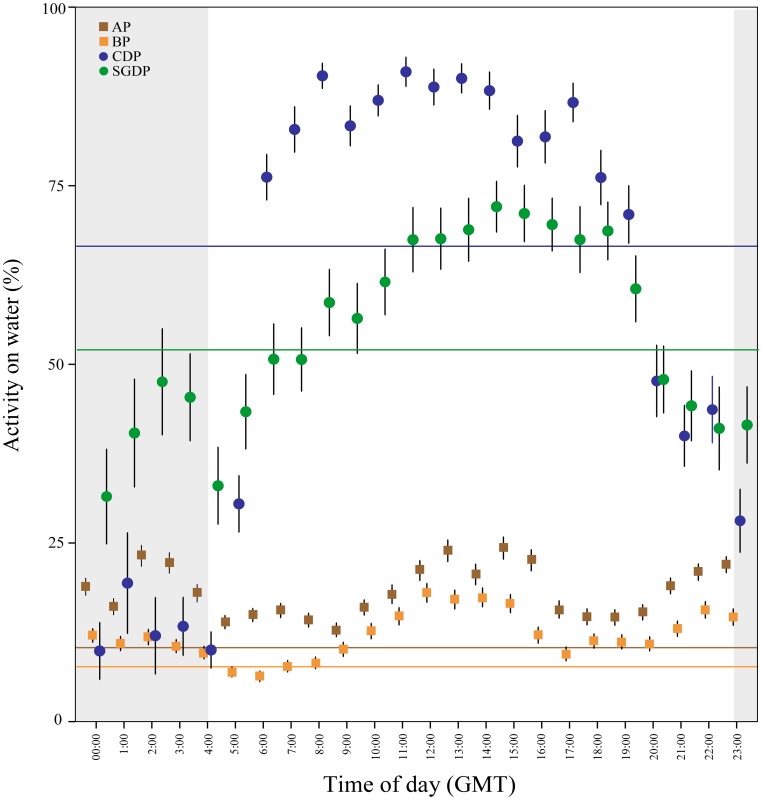
At-sea activity patterns. Mean daily percentage of time spent on water ± SE of 12 Antarctic prions (AP), 12 blue petrels (BP), 9 common diving petrels (CDP), and 8 South Georgian diving petrels (SGDP) tracked using immersion loggers during incubation at Bird Island, South Georgia in summer 2010/11. Shaded areas indicate darkness. The mean value of waveform for each species is indicated with a horizontal line (different colour for each species).

Two main peaks of immersion activity were apparent: a diurnal peak during the latter part of daylight (afternoon and early evening) for Antarctic prions, and a peak at midday for blue petrels. Both species also showed activity bouts during morning twilight, shortly before sunrise. The output of the waveform analysis (Fig. 6) shows a bimodal activity profile in the majority of Antarctic prions and blue petrels: there was a peak of activity indicating greater time spent on the water during evening twilight, and; both species showed a diurnal peak in immersion around midday (see Fig. 6). The amount of time spent on water was estimated by waveform values above the MESOR. This analysis showed lower amplitude values for Antarctic prions and blue petrels than for common and South Georgian diving petrels (one-way ANOVA; *p*<0.001, HSD test, *p*<0.001 for all the comparisons).

**Figure 7:**
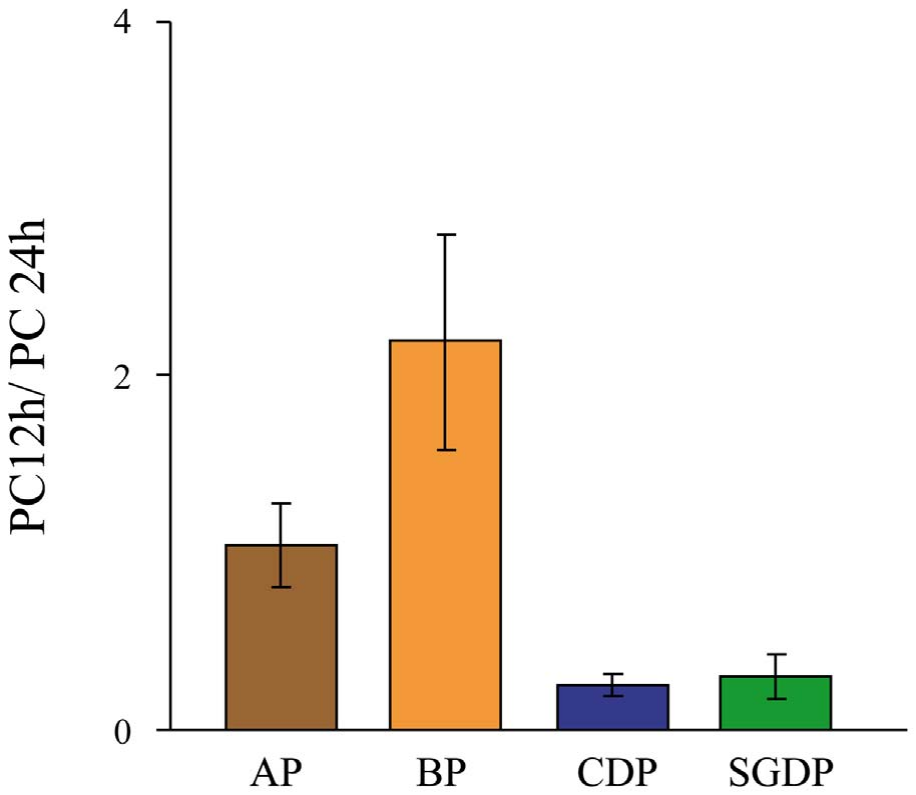
Comparing the relevance of 12 h and 24 h at-sea activity patterns. The ratio of the power content of the 12 h and 24 h harmonics obtained by Fourier analysis (mean and standard deviation), indicating the relevance of the 12 h activity bout to the 24 h pattern, for (A) Antarctic prion, (B) blue petrel, (C) common diving petrel and (D) South Georgian diving petrel.

The PC12 h/PC24 h ratio (Fig. 7) differed significantly only between blue petrel and common diving petrel (one-way ANOVA; *p*<0.05, HSD test, *p*<0.05), but not among Antarctic prion, common and South Georgian diving petrels (all pairwise tests, p>0.05). Blue petrels had a robust 12 h harmonic component relative to the 24 h component, whereas these showed similar variability in Antarctic prions. Common and South Georgian diving petrels showed minor variability in the 12-h activity pattern in proportion to the 24 h harmonic.

**Figure 8:**
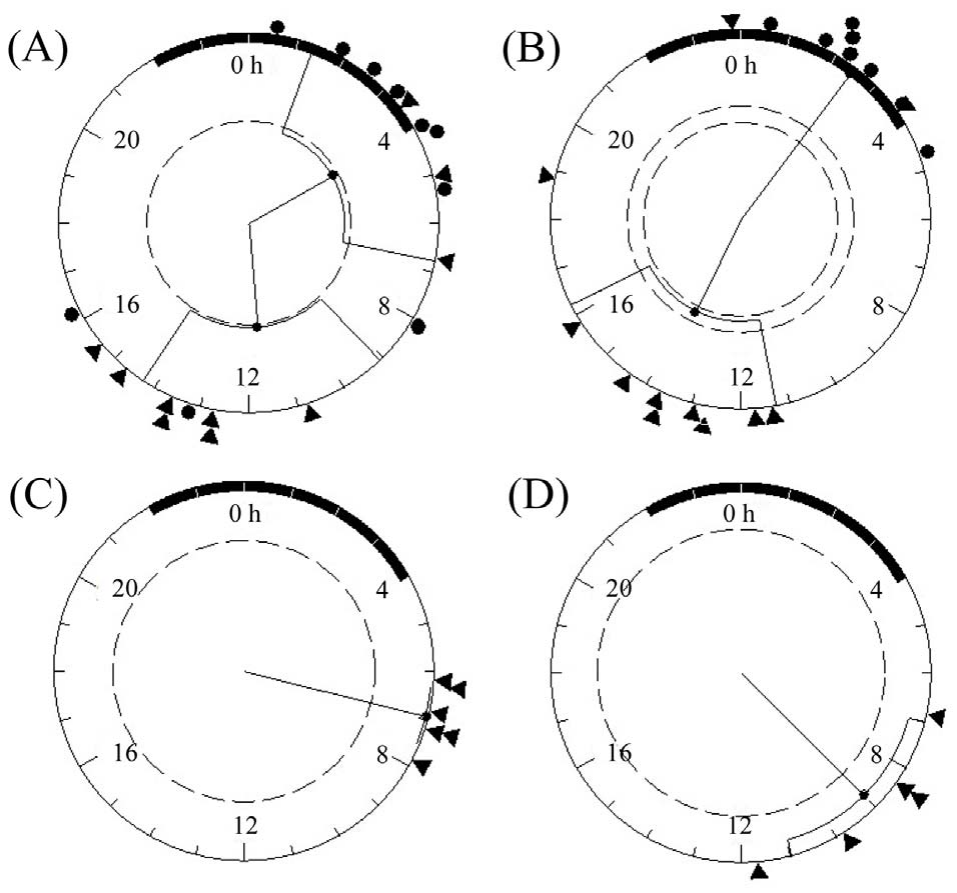
Temporal differences in the main and secondary at-sea activity bouts. Outputs from Rayleigh z-tests used to evaluate clustering of the times of the main and secondary activity bouts (indicated by triangles and circles, respectively) estimated from waveforms of Antarctic prions (A) and blue petrels (B) or of the main bouts only of common diving petrels (C) and South Georgian diving petrels (D). Tick marks from 0–24 represent the hours of the day, the black semicircle depicts night (sunrise at 4∶00 GMT, sunset at 22∶00 GMT). The dotted circumference defines the threshold value for a significant *r* vector (p<0.05). The mean vector for individual values is indicated with confidence intervals.

The main (for all species) and secondary activity bouts (for Antarctic prions and blue petrels) were studied using circular statistics (Rayleigh z-tests are shown in Fig. 8). For Antarctic prions and blue petrels, a significant clustering was apparent for the main immersion activity bout that occurred during daytime (Antarctic prions: *r* = 0.55, *r*(0.05) = 0.53; mean vector phase: 11.7-h [9.1-h - 14.3-h]; blue petrels: *r* = 0.54, *r*(0.05) = 0.52; mean vector phase: 13.8-h [11.3 h –16.3 h]). The nocturnal, secondary immersion activity bout, was only significantly clustered for blue petrels (*r* = 0.95, *r*(0.05) = 0.61; mean vector phase: 2.5-h [1.45.h - 3.4-h]) but not for Antarctic prions, since the main immersion activity bout occurred during darkness in some individuals, and the secondary immersion activity bout during daytime. The onset of activity appeared clustered at around 0700 to 0900h GMT/UTC (local time is GMT/UTC - 2h) in individuals of both common and South Georgian diving petrels, generating significant clustering in the Rayleigh z-tests (for common diving petrels: *r* = 0.99, *r*(0.05) = 0.67, mean vector phase: 6.9-h [6.3-h - 7.5-h]; for South Georgian diving petrels: *r* = 0.91, *r*(0.05) = 0.84, mean vector phase: 9.0-h [7.0 h –11.0-h].

### Stable Isotope Ratios

**Figure 9:**
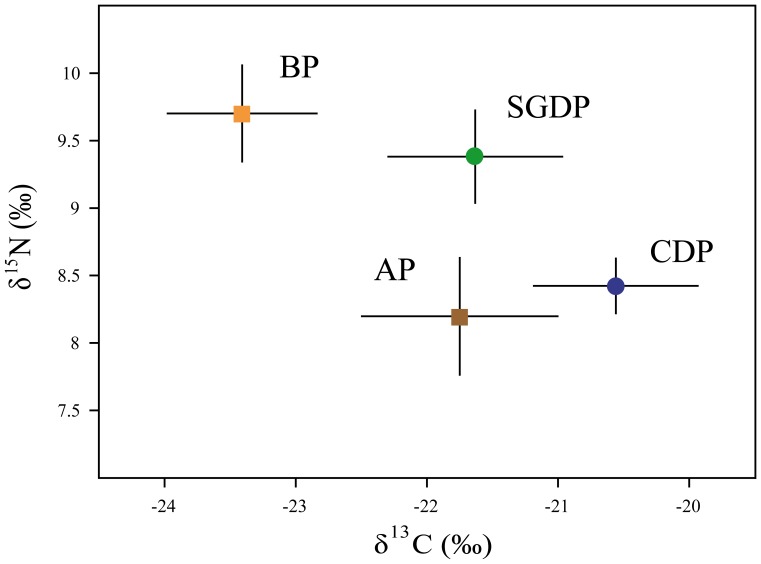
Isotopic niche differences. Mean ± SD of δ^15^N and δ^13^C values in red cells of four species of small petrel sampled during incubation at Bird Island, South Georgia in summer 2010/11. Species abbreviations: AP (Antarctic prion), BP (blue petrel), CDP (common diving petrel) and SGDP (South Georgian diving petrel). All n = 15.

δ^13^C values differed significantly among species (ANOVA test, *F*_3, 59_ = 67.81 p<0.0001; Fig. 9); all 4 pairwise species comparisons were also significant (Tukey test: all p<0.05). δ^15^N values also differed significantly among species (*F*_3, 59_ = 50.93, p<0.0001; Fig. 9). Mean δ^15^N was significantly higher in blue petrel and South Georgian diving petrel than in common diving petrel and Antarctic prion (Tukey test, p<0.0001).

### Flight Performance Metrics

**Figure 10:**
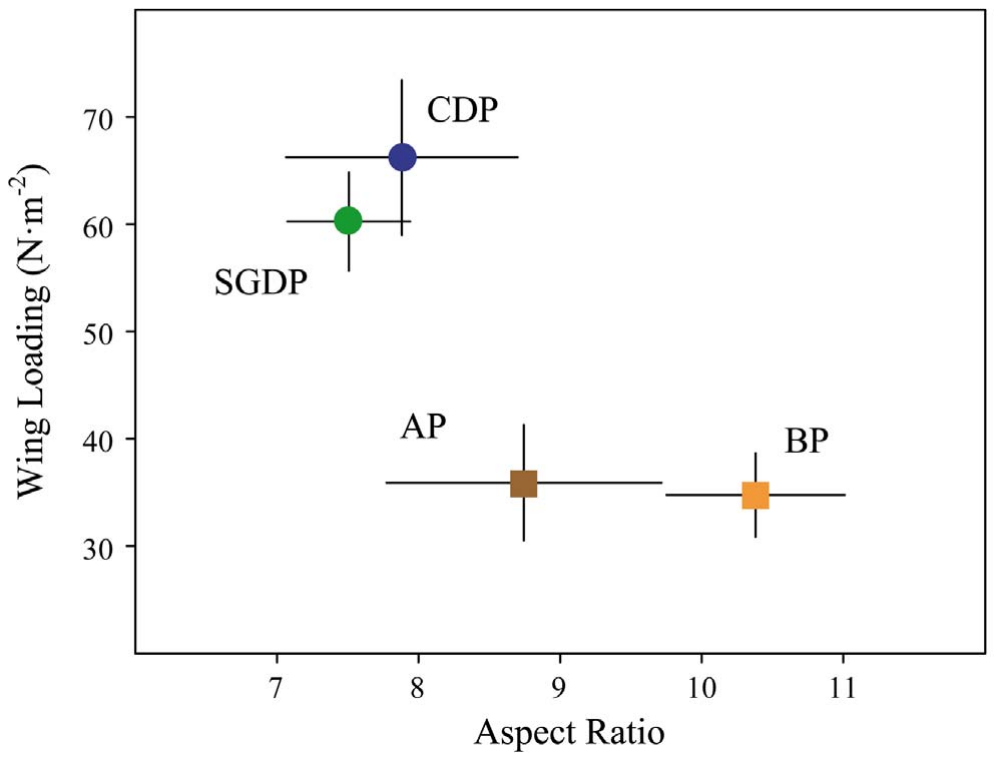
Flight performance descriptors. Mean ± SD of wing loading and aspect ratio of four species of small petrel measured during incubation at Bird Island, South Georgia in summer 2010/11. Species abbreviations: AP (Antarctic prion), BP (blue petrel), CDP (common diving petrel) and SGDP (South Georgian diving petrel). All n = 15.

Mean body mass ± SD was 186.7±13.8 g, 163.5±14.8 g, 146.8±5.5 g and 123.8±12.4 g for blue petrel, Antarctic prion, common diving petrel and South Georgian diving petrel, respectively, and differed significantly among species (*F*_3, 59_ = 71.01, p<0.0001; all four Tukey pairwise comparisons, p<0.05). Wingspan showed a similar pattern (74.01±2.46 cm, 62.54±2.37 cm, 41.39±1.54 cm and 38.83±1.17 cm for blue petrel, Antarctic prion, common diving petrel and South Georgia diving petrel, respectively; *F*_3, 59_ = 1114.61, p<0.0001; Fig. 10). Wing loading also differed among species, and was significantly greater in common and South Georgian diving petrels than in Antarctic prion and blue petrel (*F*_3, 59_ = 136.09, p<0.0001; Tukey tests, both p<0.0001; Fig. 10). Aspect ratio showed a different pattern; highest in blue petrel followed by Antarctic prion, and low in both common and South Georgian diving petrel (*F*_3, 59_ = 44.63, p<0.0001; Tukey tests, both p<0.0001; Fig. 10).

## Discussion

By combining information obtained from tracking devices, stable isotope analyses and an assessment of flight performance, we provide a very detailed investigation of ecological segregation in four species of sympatric small petrels that are major consumers in Southern Ocean ecosystems. Given that these species are all zooplanktivores of broadly similar body size, and so potentially dependent on similar trophic resources, their coexistence should be possible only if there is a degree of segregation in one or more aspects of their ecological niches in order to avoid competition [6], [24]. The diving, at-sea diel activity and distribution data from the GLS-immersion loggers and TDRs confirm a combination of horizontal, vertical and temporal segregation in terms of their foraging behaviour, at least during the incubation period (summarised in Table 2). Analysis of stable isotope ratios in blood revealed that all species had different trophic niches. In addition, the morphological analyses indicated considerable divergence in terms of flight performance. The implications of these results for understanding resource partitioning in colonial petrels in particular, and seabirds in general are discussed below.

**Table 2:** Summary of the evidence in the present study for interspecific overlap/segregation in each ecological niche axis between Antarctic prion (AP), blue petrel (BP), common diving petrel (CDP) and South Georgian diving petrel (SGDP).

	Horizontal axis (spatial distribution)	Vertical axis (diving distribution)	Temporal axis (timing of foraging)	Isotopic axis (distribution and trophic level)
**AP**	Limited overlap with SGDP, CDP	Extensive overlap with BP(shallow diver)	Nocturnal and diurnal; overlapwith BP	δ^13^C: Segregated δ^15^N: Similar to CDP
**BP**	Largely segregated	Extensive overlap with AP(shallow diver)	Nocturnal and diurnal; overlapwith AP	δ^13^C: Segregated δ^15^N: Similar to SGDP
**SGDP**	Overlap with CDP & partialoverlap with AP	Largely segregated(very deep diver)	Diurnal; overlap with CDP andpartial overlap with AP & BP	δ^13^C: Segregated δ^15^N: Overlap with BP
**CDP**	Overlap with SGDP & AP	Largely segregated (deep diver)	Diurnal; overlap with SGDP and partial overlap with AP & BP	δ^13^C: Segregated δ^15^N: Similar to AP

Although the at-sea distributions of all four species of small petrel were largely south of the Antarctic polar front, with the exception of the two diving petrels, there was clear spatial segregation. During incubation, Antarctic prions foraged to the west of the South Georgia archipelago in two core areas, one close to the colony and the other much further to the south. These results are consistent with a previous study based on stable isotopes, which suggest that during chick-rearing, Antarctic prions at the Kerguelen archipelago alternated the use of foraging areas close to the breeding colony with distant areas [27]. Indeed, the very broad range of δ^13^C values in Antarctic prions blood-sampled at Bird Island during chick-rearing in a previous season [23] suggests that birds continue to feed in waters ranging from the Antarctic polar front to high latitudes throughout the breeding period. Although there was some overlap at sea between Antarctic prions and blue petrels, the latter predominantly used a more extensive area to the south and southwest of South Georgia. This spatial segregation would explain the differences recorded in a conventional dietary assessment in the 1970s at South Georgia, which indicated that although Antarctic krill was the predominant prey, blue petrels consumed more fish and Antarctic prions consumed more copepods [25].

Core foraging areas of the two diving petrel species showed much greater overlap, reflecting a reliance on short foraging trips to the west of the colony. This contrasts with previous conclusions based on ship-based observations around South Georgia and stable isotope data from a study conducted in the Kerguelen archipelago suggesting that these two species showed a degree of spatial segregation [28], [44]. However, diving petrels are notoriously difficult to identify to species level at sea [13]. In any event, the broad-scale differences among the four species are consistent with the known biogeography of their main prey. The abundance of copepods, the predominant crustacean consumed by the two diving petrels [26], [28], is high in the waters around South Georgia [45], whereas Antarctic krill and other euphausiids, the main components in the diet of Antarctic prion and blue petrel [25], are more abundant to the southwest, and south to the ice edge [46].

The spatial segregation revealed by tracking data corresponds with species-specific differences in wing morphology and various aspects of flight performance. A low wing-loading and high aspect ratio is effective for long-distance flight in windy conditions, which presumably enables Antarctic prions and blue petrels to exploit distant waters in the central and southern Scotia Sea. In contrast, the high wing-loading and low aspect ratio of both diving petrels is better suited to short-distance foraging close to the colony. Moreover, the compact shape and short wings of the diving petrels confer an advantage in terms of diving capability. Indeed, common and South Georgian diving petrels dive deeper, spend more time diving and dive more frequently than Antarctic prions and blue petrels. Although diving is much more energetically-expensive than in-flight foraging, diving petrels are clearly much better adapted than the other two species to exploit the vertical distribution of their main food resources, which are copepods and euphausiids [26], [28]. Thus, foraging in much deeper water reduces inter-specific competition for food with the hugely abundant Antarctic prion, despite the overlap in (horizontal) distribution to the west of South Georgia.

Although diving petrels showed higher diving capabilities than Antarctic prions and blue petrels, they did not dive as deep as suggested from previous studies using capillary-tube depth gauges (common diving petrels = 30–40 m; South Georgian diving petrels = 25 m; [28], [30]). This difference can probably be attributed to the lower accuracy of capillary tubes, which tend to overestimate maximum depth because of the accumulation of moisture within the tubes and the impact of entering the water at speed [47], [48]. In addition, despite previous studies suggesting that common diving petrels dived deeper than South Georgian diving petrels [28], [30], here we found the opposite pattern. Aspect ratio tended to be lower in South Georgian diving petrels, which corresponds with their somewhat deeper dives and longer maximum dive durations. The contrast with previous studies could result from a seasonal shift in the vertical distribution of prey because the deployment periods for each species were a month apart, reflecting the difference of several weeks in timing of breeding. However, diet differences are maintained in the period when both species are rearing chicks, suggesting consistent differences in the way they exploit the water column [26]. In any event, reduced overlap in the water column would reduce inter-specific competition, which may be a key mechanism leading to ecological segregation given the overlap in horizontal distribution.

In addition to horizontal and vertical segregation among sympatric seabirds, differences in timing of foraging may also be an important mechanism reducing inter-specific competition [8], [10]. Although several species of Procellariidae are thought to forage routinely at night [13], we recorded this only for Antarctic prions and blue petrels - both showed more frequent dives during darkness, in contrast with the two diving petrel species which restricted their immersion and diving activity almost exclusively to daylight hours. This temporal segregation could be explained by a combination of differences in diving capability, visual acuity and the vertical migration of the key crustacean prey. Since the diving capability of Antarctic prions and blue petrels is low, it is probably uneconomic for them to dive until darkness, when the diel vertical migrations of Antarctic krill and other euphausiids brings them into the upper layers of the water column [25]. The increase in the duration of immersion events at night probably indicates an increase in dive rate or the use of a sit-and-wait feeding strategy. Similar behavioural adjustments have been observed in a wide range of marine organisms [8], [49]–[51]. This behaviour would be helped by the good night vision of prions and blue petrels [52]; in contrast, diving petrels have much poorer visual acuity [53]. During daylight, Antarctic prions and blue petrels also showed immersion activity, probably related to surface-feeding or resting on the sea surface [27], [29].

As there is evidence for substantial variation in δ^13^C and δ^15^N values of similar organisms sampled in the Southern Ocean across spatial scales as small as <100 km and depths of <10 m [22], [45], [46], [54], we interpret our isotopic results with reference to the concurrent tracking data. Given the latitudinal variation in δ^13^C and δ^15^N in the South Atlantic [22], [45], [46], the low δ^13^C and high δ^15^N values shown in blue petrels correspond well with their use of southerly waters during incubation (Fig. 1). Indeed, based on feather isotope data, this habitat preference is maintained during the non-breeding period [55]. By comparison, the high δ^13^C values in both diving petrel species correspond with the isotopic signature of waters much closer to the breeding colony [22], [45], [46]. Although δ^13^C in a small number of Antarctic prions was fairly low, indicating the use of Antarctic waters, in the majority the values were more similar to those of diving petrels, suggesting most prey were consumed in nearby areas [27]. Although foraging areas largely overlapped, and previous conventional dietary assessment suggested they consume broadly similar resources [26], South Georgian diving petrel showed considerably higher δ^15^N and δ^13^C values than common diving petrel. However, this difference in isotopic niche is actually intuitive, as copepods and other crustaceans close to South Georgia show an enrichment in δ^15^N by depth [22], and the South Georgian diving petrels dived more deeply.

In conclusion, the present study indicated that although there was a degree of overlap in some aspects of foraging behaviour, overall the four taxa operated in very different ecological space despite breeding in close proximity on land. It therefore provides important insights into the mechanisms allowing coexistence of these very large populations of predators. A key finding was that the degree of segregation varied according to which of the four different axes was being examined – a theme central to Hutchinson’s n-dimensional hypervolume model of the ecological niche. From a technical perspective, our study revealed the advantages of integrating results from the simultaneous use of miniaturized devices, stable isotope analyses and morphological measures to address foraging segregation and coexistence of closely related, sympatric taxa. More investigations are now required to improve our knowledge of how these species might mitigate competition during other breeding stages and, in particular, during the non-breeding season when there are no central-place constraints. In addition, given the evidence that Antarctic krill abundance has dramatically decreased in the Scotia Sea during the last decade [56], it will be useful to repeat such studies in the future to determine the repercussions of such a major resource decline for the structure and functioning of this and other krill-dependent communities.
